# Erratum for Steigmann et al., “Effects of immunoproteasome inhibition on acute respiratory infection with murine hepatitis virus strain 1”

**DOI:** 10.1128/jvi.01370-26

**Published:** 2026-09-15

**Authors:** Jacob C. Steigmann, Xiaofeng Zhou, Lauren N. Suttenberg, Irha Salman, Zainab F. Rehmathullah, Jason B. Weinberg

## ERRATUM

Volume 98, no. 12, e01238-24, 2024, https://doi.org/10.1128/jvi.01238-24. Materials and Methods, 6th paragraph: “10^5^ plaque-forming units” should read “5 × 10^3^ plaque-forming units.”

